# Progress in Phototherapy

**Published:** 1903-05-23

**Authors:** 


### Progress in Phototherapy.

(*Concluded from page* 119.)

That the use of the $x$-rays may be followed by the disappearance of malignant tumours is the belief of Dawson Turner,[15] although hitherto only improvement has been noticed in his cases. This was most noticeable in breast cases. A sarcoma of the parotid temporarily disappeared after 20 or more exposures, and a recurrent scirrhus of the breast after eight. He lays emphasis on the diminution of pain attending the use of the rays, the loosening of adhesions, and the healing of ulcerating and sloughing surfaces.

Marsh[16] thinks that metastases may occur more rapidly than normally with the use of the rays in sarcoma. In a case under his notice where a recurrent melanotic sarcoma was treated by the rays, a blood-count was made subsequently to each application. The leucocytes were found to have diminished markedly (22,500 to 12,000), and the hæmoglobin to be increased. The patient seemed to be worse, more tumours appeared, and those present were increased in size and were more deeply pigmented: pain was also great from one of the tumours. According to this writer the rays tend to lessen the natural protection of the body against the disease.

Two out of 16 cases of "epithelioma" failed to derive benefit from the $x$-rays in the hands of Johnson and Merrill[17] other than relief from pain and diminution of discharge. Ten cases were cured, four showed improvement, of which three give promise of ultimate recovery. Seven inoperable carcinomata only experienced relief from pain and possibly some prolongation of life. In their opinion, lupus vulgaris can be cured while fibromata show no change.

Turnure[18] considers that the $x$-rays should be used only on superficial disease processes. He has satisfactory record of 82 out of 94 cases treated with the rays, and his experience is that apparently in some carcinomata the progress of the disease is hastened. No satisfactory result occurred in two cases of sarcoma other than relief from pain for a few weeks. Two cases of tubercle of the larynx were unaffected. Two cases of lupus were cured. Of three cases of erythematous lupus after over four months of treatment two did extremely well, one showed only slight improvement. Five cases of chronic eczema of the leg were improved, apparently as a result of irritation produced; the itching ceased very promptly after the treatment.

A practical suggestion for the use of the rays in malignant disease is given by Carl Beck,[19] who recommends the fearless use of this agent without the use of masks, "so that the malignant cells which are distributed in the vicinity may not be protected from the rays." He considers that the irradiation should be carried out to a dermatitis and prolonged after the case seems well. There is a use for the rays as a prophylactic agency after disease has been removed surgically.

The inflammation caused by $x$-rays upon rodent ulcers can be greatly increased by irritants[20]—*e.g.*, 1-20 carbolic—and Mayou[21] points out that a local injury to a part under treatment by the rays may cause sloughing of the skin. Dilatation of the blood vessels occurs at the site of raying, and this is more or less permanent. In rodent ulcer the cells themselves act as irritants to the normal skin around and beneath. Increased irritation leads to the masses being thrown off as sloughs. Underneath are isolated rodent cells, which become encircled by leucocytes which endeavour to destroy the rodent cells or to so encapsulate them as to cut off nutrition. Degeneration takes place from without inwards, the epithelial cells become vacuolated, the granules in them are increased, and make their way through the cell walls and finally disappear. The chromatin gradually ceases to take up the stain, and nothing is left but an empty cell wall which is apparently devoured by the leucocytes.

Stephenson and Walsh[22] first reported the possibility of curing trachoma or "granular lids" with the $x$-rays or high-frequency currents, and Mayou substantiates this as far as the $x$-rays are concerned, though the methods differ somewhat. The former used in two cases an average exposure of 10 minutes at 8 inches distance, and found that 17 sittings were sufficient in one, and 8 in the other case. In another case they used the high-frequency brush applied through a vulcanite electrode, and cured a severe case in 22 applications. The treatment is painless, rapid and simple with both apparatuses, and thus points in their belief to some common factor, possibly a brush discharge, visible in the one, invisible in the other. Mayou uses a moderately soft tube, with 6 ampère current, a distance of 9 inches and a two minutes' exposure without protecting the eye. He treats the case on four to six successive days and then gives a week's interval; if no reaction ensues the treatment is recommenced twice a week till photophobia warns him of a reaction. By this time the granules have begun to disappear, and exposures are continued cautiously till they are gone. The result is excellent, the conjunctiva being intact and the scar so supple as to leave no deformities behind. He also points out that pannus is affected advantageously by the rays, often clearing with great rapidity and more thoroughly; even dense corneal opacities may be improved.

Foreign bodies may be localised very successfully in the globe of the eye by means of the $x$-rays. Mayou[23] has found and localised 13 out of 16 by these means. He emphasises several points in the procedure: (1) The attempt should be repeated if no foreign body be found at first. (2) Separate plates should be used, and not the same, to avoid blurring of the image. (3) Care must be taken not to let the cross-localising wires fall within the shadow of the orbit. (4) The size can be estimated as well as the position. (5) And by the stereoscope the shape can be discovered.

Pusey[24] justifies the use of the *x*-rays in cases of acne by pointing out that these rays have an inhibitory influence on the formation of pus in the skin, and they produce atrophy of cutaneous follicles. While treating a girl, aged 22, for hypertrichosis he produced an erythema on the face which was affected by acne. This affection was completely relieved, and during the year she was under treatment, off and on, he found no tendency to recur in the acne. Sycosis has also yielded to the same treatment in his hands.

Mycosis fungoides has been cured by the *x*-rays in the hands of Allan Jamieson.[25] The patient was a woman, aged 54, and the tumours and an eczematoid eruption disappeared, improvement being observed on the third day after commencement.

There is some difference of opinion as to the result upon the epithelial cells after treatment with the *x*-rays. Ellis[26] reports the histological findings in four cases as follows:—(1) Necrosis of cells and trabeculæ of varying degree; (2) increase of elastic tissue in three cases, examined both before and after exposure; (3) fewer areas of lymphocytic infiltration in one case after exposure, about equal numbers in the others; (4) a tendency to occlusion of vessels by deposits upon their inner surfaces marked in some cases; (5) Practically entire absence of infiltration by polymorpho-nuclear leucocytes. He thinks that probably necrosis is not absolutely dependent upon the endarteritis, but that both result from the same changes. Apparently there are no bactericidal tendencies in the rays, and he urges curetting diseased areas before raying.

Shattock,[27] on the other hand, has examined a tumour which had been under the rays for three months and found no changes attributable to the rays, either macro- or microscopically. Although clinically there was some diminution in the size of the tumour and it became more moveable on subjacent parts, there was no degeneration or cell lysis discovered. He thinks that the clinical signs were due to disappearance of sub-inflammatory œdema. In all there had been 50 daily exposures to the rays of 10 minutes to three-quarters of an hour; but the skin at no time showed any reaction.[28]

Taylor,[29] again, considers that the rays have a specific action upon degenerated epithelium; but in lupus he considers that the remedial influence is attributable to the burning action and to no specific influence.

The value of the rays in the localisation of cerebral tumours is attested by Mills,[30] who found that a skiagram of the head of a girl, aged 21, who had symptoms of cerebral tumour on the right side, showed an abnormal shadow over the Rolandic area. Under the guidance of this skiagram an operation was performed, but so much hæmorrhage was encountered that further steps had to be postponed. Later, after clamping the carotids, the tumour was removed as an encapsulated ovoid, 3 inches in length, and was found to be a spindle-celled sarcoma. The patient, unfortunately, died from shock.

A. B. Johnson[31] has taken 200 radiographs for stone, and although in almost all the cases the stones were found at operation they only appeared on the plate in some 30 cases. Uric acid pure is very permeable to the rays, though an admixture of only 10 per cent. calcic phosphate caused the shadow to become distinct. In one case a small stone of calcium oxalate showed distinctly, though it only weighed $1\frac{1}{4}$ grs.

Lewis Cole[32] shows us how many skiagraphic errors are to be avoided; and points out how, by change in position of an *x*-ray tube, the resulting skiagram can be made to indicate either overlapping of fragments in a fracture or separation of them. He showed a skiagram of his own arm taken in such a way as to show apparently a Colles's fracture where none existed.

[15] Scotti-h Med. and Surg. Jour., December, 1902. [16] Amer. Jour. Med. Sci., February. [17] Amer. Jour. Med. Sci., February. [18] Med. Rec., Feb. 7, 1903. [19] Med. Rec., Jan. 31, 1903. [20] Brit. Med. Jour., Dec. 6. [21] Lancet, Feb. 28. [22] Lancet. Jan. 24. [23] Lancet, Feb. 28. [24] Brit. Med. Jour., April 18 [25] Scottish Med. and Surg. Jour., December 1902. [26] Amer. Jour. Med. Sci., January 1903. [27] Lancet, Dec. 8. [28] Brit. Med. Jour., Dec. 6. [29] Amer. Jour. Med. Sci., March 1903. [30] Brit. Med. Jour., Jan. 24. [31] Med. Rec., Jan. 10. [32] Med. Rec., Jan. 10.

